# SMARCA4-Deficient Carcinoma of Unknown Primary Presenting with Fatal Paraneoplastic Hypercalcemia in a Heart Transplant Recipient: First Report in a Male Patient

**DOI:** 10.1155/2017/9403467

**Published:** 2017-10-11

**Authors:** Abbas Agaimy, Deike Strobel, Thomas Strecker

**Affiliations:** ^1^Institute of Pathology, Friedrich-Alexander-University Erlangen-Nuremberg, University Hospital of Erlangen, 91054 Erlangen, Germany; ^2^Department of Internal Medicine-1, Friedrich-Alexander-University Erlangen-Nuremberg, University Hospital of Erlangen, 91054 Erlangen, Germany; ^3^Department of Cardiac Surgery, Friedrich-Alexander-University Erlangen-Nuremberg, University Hospital of Erlangen, 91054 Erlangen, Germany

## Abstract

Small cell carcinoma of the ovary, hypercalcemic type (SCCOHT), is a rare SMARCA4-driven aggressive malignancy of young age characteristically associated with paraneoplastic hypercalcemia. Comparable neoplasms/presentations have not been reported in males. A 39-year-old male heart transplant recipient (HTX 40 months previously) presented with multiple liver nodules and hypercalcemic crisis. The serum parathyroid hormone-related protein (PTHrp) was significantly elevated (241 pg/ml; reference value < 57). Liver biopsy showed poorly differentiated partially rhabdoid neoplasm expressing pancytokeratin, CK20, and focally GATA3, SATB2, p63, and SALL4. The tumor cells showed dual loss of SMARCA4 and SMARCA2. He died of irreversible multiorgan failure one week after admission. To our knowledge, this is the first report highlighting the rare occurrence of paraneoplastic hypercalcemia associated with SMARCA4-deficient malignancies in males. Although the immunophenotype suggests urothelial or upper gastrointestinal tract origin, the exact primary tumor site could not be ascertained due to rapid death of the patient. SMARCA4 immunohistochemistry should be included in the workup of neoplasms associated with hypercalcemia irrespective of gender and site.

## 1. Introduction

The SWItch Sucrose Non-Fermentable (SWI/SNF) complex is a highly conserved large complex of >20 functionally closely related proteins encoded by genes located on different chromosomal regions, but functioning in a highly coordinated manner to regulate the process of chromatin remodelling and hence tightly regulate gene expression [1]. Beginning with recognition of SMARCB1/INI1 (mapped to chromosome 22q) as the driver of the aggressive pediatric malignant rhabdoid tumors decades ago [2], the role of the SWI/SNF complex as a tumor suppressor has been increasingly recognized in a variety of neoplasms originating at a wide age range in different anatomic organs. SWI/SNF-related neoplasms display diverse lines of differentiation, but generally are unified by presence of rhabdoid cell features, highly aggressive clinical course with early dissemination of the disease, and frequently rapid death [3]. Following delineation of the roles of SMARCB1 (INI1) in different neoplasms [3], SMARCA4 (BRG1) on chromosome 19p13.2 has been recently increasingly recognized as the main or sole molecular driver event in SMARCB1-intact rhabdoid malignancies including in particular the vast majority of small cell carcinomas of ovary, hypercalcemic type (SCCOHT) [4]. This highly aggressive malignancy affects mainly girls and young women (<35 years) with poor outcome due to rapid disease dissemination and limited effective therapy [5]. To our knowledge, SMARCA4-driven carcinoma associated with paraneoplastic hypercalcemia has not been reported in a male before. This is the purpose of this novel case study.

## 2. Case History

**Figure 1:**
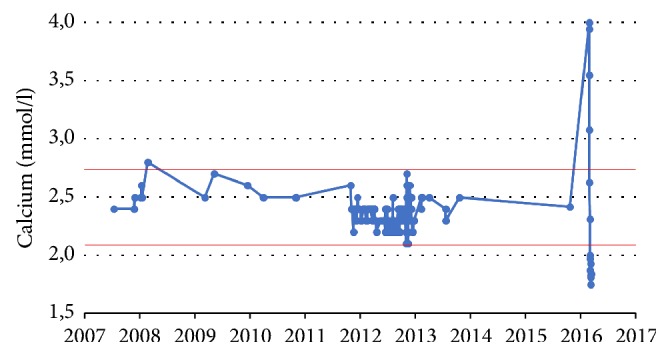
Patient's serum calcium values over the years (since his initial cardiac disease in 2007) showed significant hypercalcemia during his fatal disease.

A 39-year-old Caucasian male was readmitted to our hospital with significant hypercalcemia of 4.0 mmol/l (reference range, 2.1 to 2.7 mmol/l) (Figure 1). At the time of admission, his serum parathyroid hormone-related protein (PTHrp) was significantly elevated (241 pg/ml; reference value < 57). He reported progressive malaise, general weakness, repeated emesis, and colicky pain in the upper right abdomen. Forty months previously, he had undergone heart transplantation for end-stage heart failure as a result of dilative cardiomyopathy (DCM) for approximately five years. Routine follow-up posttransplantation endomyocardial biopsies (EMB) demonstrated no evidence of graft rejection; the last routine EMB was one week before his recent admission. At this time, transthoracic echocardiography (TTE) revealed an almost normal left and right ventricular function with an ejection fraction (EF) of 50%, well-functioning heart valves, and no evidence of pericardial effusions. At the latest admission, the blood analysis showed elevated inflammation parameters: C-reactive protein (CRP) was 164.7 mg/l (reference range < 5 mg/l); procalcitonin (PCT) was 7 ng/ml (reference range < 0.5 ng/ml). The liver function test showed elevated glutamate-oxalacetate-transaminase (GOT) of 98 U/l (reference range < 50 U/l) and bilirubin was 3.4 mg/dl (reference range < 1.1 mg/dl). Following thorough volume substitution and antibiotic therapy the patient's health status improved temporarily. Abdominal ultrasonography demonstrated hepatomegaly with multiple liver nodules. Subsequently, computed tomography (CT scan of abdomen and thorax) confirmed disseminated heterogeneous liver lesions suspicious of metastases. In addition, multiple enlarged mediastinal, hilar, and para-aortic lymph nodes were interpreted as suspicious of metastases as well but there was no evidence of other intra-abdominal or intrathoracic primary tumor.

During the next few days, the patient's condition deteriorated rapidly. Hemofiltration was necessary in order to reduce his increasing calcium values and to obtain sufficient urinary clearance. The antibiotic regime was adjusted. Nevertheless, the patient developed high pyrexia (40 centigrade), the CRP increased to 190.3 mg/l, the PCT to 54.2 ng/ml, the GOT to 754 U/l, and bilirubin to 11.4 mg/dl indicating liver failure. Echocardiography showed severely impaired ventricular function (EF dropped to 10%). Despite maximum interdisciplinary therapy, the patient died of supervening multiorgan failure. An autopsy was not performed.

## 3. Materials and Methods

The biopsy specimen was fixed in buffered formalin and embedded for routine histological examination. Immunohistochemical studies were performed on 3-*μ*m sections cut from paraffin blocks using a fully automated system (“Benchmark XT System”, Ventana Medical Systems Inc, 1910 Innovation Park Drive, Tucson, Arizona, USA) and the following antibodies: pancytokeratin (clone AE1/AE3, 1 : 40, Zytomed, Berlin, Germany), vimentin (V9, 1 : 100, Dako, Hamburg, Germany), CK7 (clone OV-TL, 1 : 1000, Biogenex), CK20 (clone Ks20.8, 1 : 50, Dako), TTF-1 (clone 8G7G3/1, 1 : 500 dilution, Zytomed), CDX2 (clone CDX2-88, 1 : 100, BioGenex), HepPar-1 (clone OCH1E5, 1 : 200 dilution, Dako, Glostrup, Denmark), Arginase-1 (clone SP156, lot 1426205A, ready to use, Cell Marque, Rocklin, CA, USA), Glypican-3 (clone 1G12, 1 : 200, Zytomed), SALL4 (clone 6E3, ready to use, Cell Marque), AFP (polyclonal, 1 : 20, Dako), ERG (clone EPR3864, prediluted/ready to use, Ventana Medical Systems), PAX8 (rabbit polyclonal, 1 : 50, Cell Marque), GATA3 (clone L50-823, 1 : 1000, DCS), p63 (clone SFI-6, 1 : 100, DCS), Uroplakin II (clone AU1, 1 : 10, Zytomed), SATB2 (clone EPNCIR130A, 1 : 200, Abcam), MLH-1 (clone ES05, 1 : 50; Dako), MSH2 (clone G2-19-1129, prediluted; Ventana), SMARCB1 (INI1) (MRQ-27, 1 : 50, Zytomed), SMARCA2 (polyclonal antibody, 1 : 100, Atlas Antibodies AB, Stockholm, Sweden), SMARCA4 (anti-BRG1 antibody, clone EPNCIR111A, 1 : 100, Abcam; Cambridge, UK), and ARID1A (rabbit polyclonal antibody, ab97995, 1 : 100; Abcam) according to the manufacturer instructions.

Assessment of the staining results of the SWI/SNF components was done as recently described [6]; that is, only the nuclei of viable tumor tissue (away from necrotic areas) were assessed. As a control, the presence of a homogeneous strong nuclear staining of stromal fibroblasts, inflammatory cells, vascular endothelial cells, or normal epithelial cells in the background was a prerequisite for assessable staining in the tumor. Three staining grades were defined:* intact* (strong staining in the neoplastic cells that is similar to normal background cells),* lost* (indicating unstained neoplastic cell nuclei as opposed to strong staining in normal cells), and* reduced* (if very weak but still discernible as opposed to strong staining in normal cells).

## 4. Results

### 4.1. Pathological Findings

**Figure 2:**
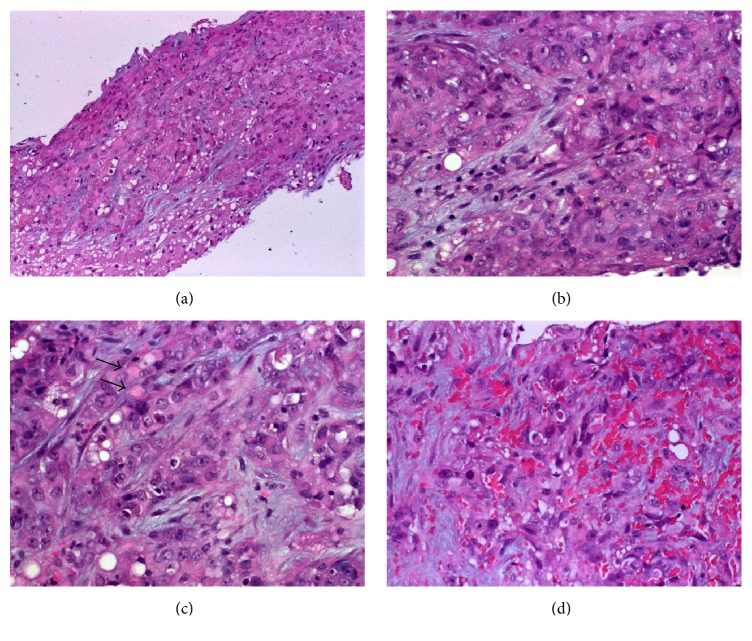
(a) Liver biopsy showed tumor tissue diffusely infiltrating the liver parenchyma (normal liver is seen in lower left field). (b) Higher magnification showed anaplastic large epithelioid cells with prominent nucleoli. (c) Scattered rhabdoid inclusions are seen (arrows) as well as small cytoplasmic vacuoles possibly suggestive of abortive glandular differentiation. (d) In other areas, hemorrhage and spindling might be mistaken for a vascular neoplasm.

Histological examination showed poorly differentiated neoplasm composed of large polygonal epithelioid cells arranged into solid nests, diffuse sheets, and adenoid aggregates with occasional lumen-like differentiation (Figures 2(a) and 2(b)). The nuclei were high-grade with vesicular chromatin and prominent central nucleoli (Figure 2(c)). The cytoplasm was pale eosinophilic, granular, and occasionally contained hyaline rhabdoid paranuclear inclusions (Figure 2(c)). Focal stromal hemorrhage with spindling of the neoplastic cells mimicking a vascular neoplasm was seen (Figure 2(d)).

**Figure 3:**
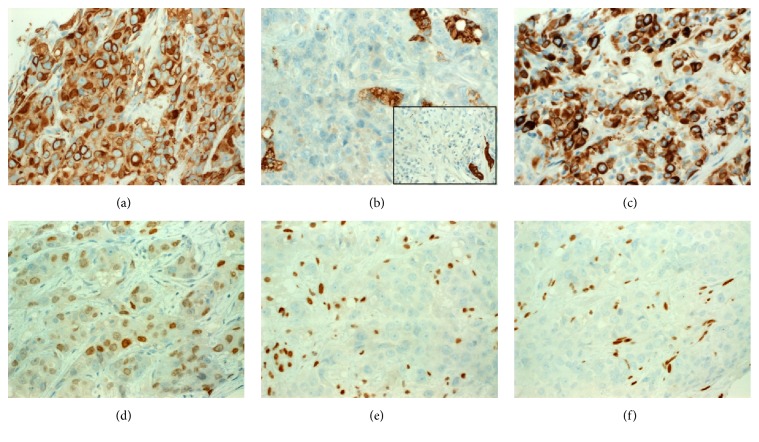
By immunohistochemistry, the neoplastic cells expressed strongly pancytokeratin with frequent paranuclear accentuation in the rhabdoid inclusions (a). The neoplastic cells were negative with HepPar-1 ((b) main image; see strongly stained hepatocytes) and CK7 ((b), inset; residual biliary tract epithelial cells are strongly stained). (c) CK20 was expressed in most of cells. (d) GATA3 was focally positive. Both of SMARCA4 (e) and SMARCA2 (f) are lost in the tumor cells (endothelial cells are strongly positive for both markers as internal control).

By immunohistochemistry, the neoplastic cells were strongly and diffusely positive for vimentin and pancytokeratin (AE1/AE3) (Figure 3(a)) but were negative for HepPar-1 (Figure 3(b)) and CK7 (Figure 3(b), inset). Of the remaining markers listed above, the tumor cells were strongly positive for CK20 (Figure 3(c)) with a variable (but mainly focal) expression of GATA3 (Figure 3(d)), SATB2, p63, and SALL4. All other markers (AFP, glypican-3, HepPar1, Arginase-1, PAX8, TTF1, ERG, and Uroplakin II, etc.) were negative. The mismatch repair proteins MLH1 and MSH2 were both intact in the tumor cells. Both of SMARCA4 (Figure 3(e)) and SMARCA2 (Figure 3(f)) were lost in the neoplastic cells but were strongly retained in the background stromal and endothelial cells. The liver showed mild steatosis; in particular, there was no evidence of liver fibrosis/cirrhosis, or chronic hepatitis. Molecular analysis of SMRACA4 could not be performed due to the limited amount of tissue available.

## 5. Discussion

Small cell carcinoma of the ovary, hypercalcemic type (SCCOHT), is a rare highly aggressive ovarian malignancy of childhood and young women (mean age: 24 yrs) [7]. Although most patients with SCCOHT present with signs and symptoms related to the underlying abdominal and/or pelvic mass, rare cases have presented with clinical features of hypercalcemia. In total, paraneoplastic hypercalcemia was recorded in two-thirds of the patients [7]. Some studies have documented elevated parathyroid hormone-related protein in the serum of affected patients as a possible explanation for the development of hypercalcemia [7]. SCCOHT is highly aggressive with half of the cases being already disseminated to intra-abdominal or other organs by the time of diagnosis. Histologically, classical or prototypical (undifferentiated small blue cells, hence the name “*small cell carcinoma*”) and large cell (rhabdoid) subtypes were recognized [7]. To our knowledge, hypercalcemic SMARCA4-deficient carcinoma has not been reported in a male before. Thus the current case is novel and especial in many aspects.

First, the patient has a history of orthotopic cardiac transplantation (HTX) for terminal heart failure resulting from dilative cardiomyopathy. Similar to other solid organ transplantations, HTX is known to be complicated by metachronous malignancies of diverse types and anatomic sites. The rate of metachronous malignancies in cardiac transplant recipients in previously published series varied greatly from 4% to 46%, based on the extent and completeness of follow-up [8]. At our center, metachronous malignancies affected 6% of patients and they developed 33 to 152 months after HTX (median, 77 months). Several studies reported a more aggressive course of posttransplant solid malignancies, possibly as a consequence of the underlying immunosuppression [8]. To our knowledge, this case represents the first report of a SWI/SNF-deficient malignancy in a post-HTX setting.

Second, the tumor was associated with unexplained hypercalcemia which has been interpreted clinically as a paraneoplastic phenomenon and led to the discovery of the tumor in conjunction with the upper abdominal symptoms. The clinical information regarding paraneoplastic hypercalcemia warranted the SMARCA4 immunostaining of the tumor (analogous to the SCCOHT) which indeed uncovered loss of this tumor suppressor in the neoplastic cells. The elevated serum level of PTHrp in this patient is likely responsible for his severe paraneoplastic hypercalcemia and indicates similar pathophysiological mechanism analogous to SCCOHT [7]. Accordingly, this case represents an index case pointing to the rare existence of an ovarian-analogue hypercalcemic SMARCA4-deficient carcinoma in males. The current case however corresponds to the so-called large cell (rhabdoid) variant of SCCOHT [7]. Of note, dual loss of SMARCA4 and SMARCA2 was confirmed in this case as a further analogy to the SCCOHT [9].

Third, this unusual case presented with several symptoms potentially attributable to graft failure but the cardiac biopsy obtained just prior to the rapid deterioration of the patient showed no evidence of rejection. Accordingly, the symptoms of the patient and the dramatic deterioration of his condition have been clinically attributed to the underlying hypercalcemic crisis. This presentation is exceptional even in cases of SCCOHT.

From a differential diagnostic point of view, posttransplant lymphoproliferative disorders (PTLD) [10], posttransplant Epstein Barr Virus- (EBV-) associated smooth muscle neoplasms of the liver [11], and primary or metastatic other solid malignancies in posttransplant setting [8] are the main consideration in our case on clinical grounds. The first two possibilities were then excluded immediately upon HE slides evaluation. Regarding the third point, it is not clear where the neoplasm in this case originated from. The tumor cells lacked any specific features of differentiation and could therefore not certainly be classified as either adenocarcinoma or squamous cell carcinoma. The immunoprofile was suggestive of adenocarcinoma of GI tract origin (being positive for CK20/GATA3 but negative with CK7 and Uroplakin II). The observed expression of p63 is not specific for urothelial carcinoma and has been reported in SWI/SNF-deficient GI carcinomas as well [6]. In addition, GATA3 is known to be expressed in a subset of upper GI tract adenocarcinomas including cholangiocarcinoma [12]. Moreover, SMARCA4 loss has been recently reported in up to 5% of non-small cell lung cancer [13] as well as in rare undifferentiated mediastinal malignancies [14] and in cases of undifferentiated/rhabdoid carcinoma of the urinary tract [15] and the kidney [16]. Based on these recent observations, ascertaining the primary tumor site in such setting is largely dependent on clinical examinations including whole body imaging (PET-CT) and endoscopic investigations.

In summary, we reported the first case of SMARCA4-deficient undifferentiated carcinoma presenting with severe fatal hypercalcemia causing rapid death of a cardiac transplant male. This uncommon presentation should be kept in mind when paraneoplastic hypercalcemia is recorded and warrants SMARCA4 immunostaining irrespective of gender and tumor site.
